# Integrated analysis of microbiome and host transcriptome revealed correlations between tissue microbiota and tumor progression in early-stage papillary thyroid carcinoma

**DOI:** 10.3389/fcimb.2025.1571341

**Published:** 2025-06-09

**Authors:** Xiuwen Tong, Xipei Chen, Chen Shen, Jiahao Pan, Xinyu Wang, Xinyun Xu, Sheng Liu

**Affiliations:** ^1^ Department of Thyroid and Breast Surgery, Second Affiliated Hospital of Naval Medical University, Shanghai, China; ^2^ Department of General Surgery, 928th Hospital of the PLA Joint Logistics Support Force, Haikou, China; ^3^ Department of Thyroid and Breast Surgery, Shanghai Fourth People's Hospital, School of Medicine, Tongji University, Shanghai, China

**Keywords:** PTC, tissue microbiome, host gene expression, microbe-gene association, microbe-cell association

## Abstract

**Introduction:**

Emerging evidences suggest that microorganisms in the tumor microenvironment play important roles in tumor occurrence and progression. However, the microbial distribution in the papillary thyroid carcinoma (PTC) tissue and its relationship with PTC are unclear.

**Methods:**

We performed 16S rRNA amplicon sequencing and RNA-Seq to characterize the tissue microbiome and transcriptome between the tumor and paracancerous tissue, respectively. The association analysis between microbes and host gene expression were conducted to screen the potential microbe-gene/cell interactions.

**Results:**

We found that the tumor tissues indeed harbored complex microbial communities, which showed significant differences in microbial and functional composition between the tumor and para-cancerous tissues. A set of differential microbial genera were identified to be significantly associated with the clinical factors, such as Planococcus enriched in tumor tissue, Limnobacter in T1a stage and Cutibacterium in N1b stage. 793 differential expressed genes were also identified, which are mainly enriched with functions related to cell-cell communication and extracellular matrix. In terms of the immune cell composition, 8 differential immune cell types were further identified, suggesting a significant immune response in PTC. Finally, association analysis identified 5 pairs of microbe-gene association and 1 pair of microbe-cell with significance, which were all involved in the tumorigenesis and tumor progression via inflammation-related pathways.

**Conclusions:**

In addition to characterizing the tissue microbiome and host gene expression in PTC patients, we further explored the roles of microbe-gene/cell interactions in PTC. The results provide candidate biomarkers for exploring the molecular mechanisms of tissue microbiome in tumorigenesis and tumor progression of PTC.

## 1 Introduction

Thyroid carcinoma (TC) is one of the most frequent endocrine malignancies, with a total of 586,202 thyroid cancer cases globally in 2020, ranking ninth in cancer incidence and fourth in all malignancies among women, three times higher than men (Sung et al., 2021). Differentiated thyroid carcinoma (DTC) accounts for more than 95% of all thyroid cancers and can be further divided into papillary thyroid carcinoma (PTC, 85%–90%), follicular thyroid carcinoma (FTC, 5%–10%), and Hürthle cell carcinoma (3%) (Pellegriti et al., 2013; Kure and Ohashi, 2021). The etiology and pathophysiology of PTCs are unknown, and the only recognized risk factor is ionizing radiation. However, there is some evidence that other variables such as family history, obesity, and environmental factors may enhance the chances of PTC (Stonell et al., 2022). It has recently been established that microbiome dysbiosis plays a significant role in the onset and progression of cancers like gastric cancer and may even alter the therapeutic response to chemotherapy and immunotherapy (Helmink et al., 2019). Recent studies have found that intestinal flora may play an anti-cancer or pro-cancer role in thyroid cancer by affecting hormone synthesis, enzyme activity, and immune response (Hu et al., 2024; Ludgate et al., 2024; Virili et al., 2024). Dysregulation of the gut microbiome was linked to thyroid cancer and thyroid nodules and corresponded with clinical indexes of thyroid function, according to Zhang et al.’s study (Zhang et al., 2019). As is well known, the thyroid gland develops from the primitive intestine, and thyroid follicles share the same endoderm as the mural cells and have some similar morphological and functional characteristics (Cellini et al., 2017). Both gastric mucosa and thyroid follicular cells concentrate and transport iodine via The Na(+)/I(−) symporter (NIS) (Portulano et al., 2014). It has also been found that intestinal flora may be involved in the development of thyroid cancer through NIS regulation (Samimi and Haghpanah, 2020). Lu et al. found that the diversity and abundance of gut microbiome were significantly reduced in TC patients and that imbalance of gut microbiome affects lipid metabolism in thyroid cancer patients, thus promoting cancer progression (Lu et al., 2022). Yu et al. performed a functional predictive analysis of functional and metabolic changes in the gut microbiome of TC patients and found that the microbiome alterations observed in TC lead to a decline in aminoacyl-tRNA biosynthesis, homologous recombination, mismatch repair, DNA replication, and nucleotide excision repair, which in turn play an important role in the development of TC (Yu et al., 2022).

Organs such as the thyroid and bladder were long thought to be sterile, and the arrival of second-generation sequencing technology has led to the discovery of a large microbiome residing in these organs and the observation and analysis of the relationship between the tumor microbiome and cancer (Alfano et al., 2016; Liu et al., 2018; Dai et al., 2021). A study by Nejman et al. analyzed bacterial lipopolysaccharide (LPS) and 16S rRNA sequencing results in seven tumor types and found that bacteria within tumors were located within cancer cells and immune cells and that bacterial composition varied by tumor type (Nejman et al., 2020). Yuan et al. found a large microbiome abundance in PTC tumor tissues, and tumor tissues of PTC patients with T1–T2 stages and T3–T4 stages had unique microbiome characteristics, with higher microbial alpha diversity in T3–T4 stages than in T1–T2 stages (Yuan et al., 2022). Dai et al. also found significant differences in microbial diversity between tumor and peritumor tissues in PTC patients, and a higher abundance of *Sphingomonas* was associated with lymph node metastasis (Dai et al., 2021). In a word, the intra-tumor microbiota was closely related to tumor development, including tumor aggressiveness and lymph node metastasis. However, the distribution of tissue microbiome and its effects on the host gene expression in the early stage of PTC are unknown. Whether the tissue microbiome is involved in tumorigenesis of PTC in the first place also remains unclear. Besides, it is expected to identify specific microorganisms that predict lymph node metastasis in the central region of patients with clinically negative PTC, to guide the implementation of prophylactic central neck lymph node dissection, to reduce complications such as vocal cord paralysis and muscle twitching associated with unnecessary prophylactic surgery, and to improve the quality of life after surgery.

Therefore, we recruited 38 patients with early-stage PTC. The tumor tissue and para-cancerous tissue samples were further subjected to 16S rRNA amplicon sequencing and whole transcriptome sequencing. We found that the tumor tissue indeed harbored a complex microbial community, which showed significant differences in microbial composition and functional composition between the tumor tissue and para-cancerous tissue in PTC. The host gene expression and immune cell composition of tumor tissue in PTC also showed similar results. A set of differential microbial taxa and differential expressed genes were further identified. On this basis, we finally identified five pairs of microbe–gene association and one pair of microbe–cell with significance, including the genus *Planococcus*, *Xanthobacter*, and *Blastcoccus*; the genes *GGCT*, *LOC102723808*, *EGFEM1P*, *PTGER1*, and *MFAP2*; and the cell type myeloid dendritic cell activated, which were all involved in the tumorigenesis and tumor progression via inflammation-related pathways. The results provide candidate biomarkers that may potentially serve as the targets for exploring the molecular mechanisms of tissue microbiome in tumorigenesis and tumor progression of PTC.

## 2 Materials and methods

### 2.1 Patients and sample collection

This study included 38 patients with PTC who underwent thyroidectomy at the Second Affiliated Hospital of Naval Medical University from September 2023 to December 2023. All PTC patients were newly diagnosed and evaluated by two pathologists who confirmed the classic PTC. Paired tumor tissues and para-cancerous tissues at least 1 cm from the tumor were gathered, frozen right away, and then kept frozen at −80°C in the refrigerator. In addition, sterile swabs were used to wipe sampling tools and surfaces for environmental samples as the negative control. Exclusion standards were as follows: (1) patients on antibiotics, probiotics, radiotherapy, chemotherapy, and biological therapy in the month before admission; (2) patients younger than 18 years old, or older than 75 years old; (3) patients with other malignancies; (4) patients who are pregnant or breastfeeding; and (5) patients with BMI>30 (obesity criteria). This investigation followed the Strengthening the Reporting of Observational Studies in Epidemiology (STROBE) reporting guideline for cohort studies. In compliance with the Helsinki Declaration, this study was approved by the ethics committee of the Second Affiliated Hospital of Naval Medical University. Written informed consent was obtained from all participants. Moreover, we collected clinicopathological information on patients’ sex, diagnosis, age, recurrence risk, and pathology TNM stage. Recurrence risk was determined based on the 2015 American Thyroid Association (ATA) risk stratification system. TNM staging was determined based on the 8th edition of the American Joint Committee on Cancer staging system.

### 2.2 DNA extraction and 16S rRNA sequencing

Total genomic DNA samples were extracted using the OMEGA Soil DNA Kit (M5635-02) (Omega Bio-Tek, Norcross, GA, USA), following the manufacturer’s instructions, and stored at −20 °C prior to further analysis. The quantity and quality of extracted DNAs were measured using a NanoDrop NC2000 spectrophotometer (Thermo Fisher Scientific, Waltham, MA, USA) and agarose gel electrophoresis, respectively. PCR amplification of the bacterial 16S rRNA genes V3–V4 region was performed using the forward primer *338F (5’-ACTCCTACGGGAGGCAGCA-3’)* and the reverse primer *806R (5’-GGACTACHVGGGTWTCTAAT-3’)*. Notably, the electrophoresis profiles of two negative control samples showed no discernible DNA amplification peaks between the low-molecular-weight (LM, ~200 bp) and unknown-molecular-weight (UM, ~5000 bp) regions, suggesting the extremely low or absent microbial load in the sampling environment (**Supplementary Figure S1**). Therefore, the quality control samples were not further processed for library construction and sequencing.

Sample-specific 7-bp barcodes were incorporated into the primers for multiplex sequencing. The PCR components contained 5 μL of buffer (5×), 0.25 μL of Fast Pfu DNA Polymerase (5U per μL), 2 μL (2.5 mM) of dNTPs, 1 μL (10 μM) of each forward and reverse primer, 1 μL of DNA template, and 14.75 μL of ddH2O thermal cycling consisted of initial denaturation at 98 °C for 5 min, followed by 25 cycles consisting of denaturation at 98 °C for 30 s, annealing at 53 °C for 30 s, and extension at 72 °C for 45 s, with a final extension of 5 min at 72 °C. PCR amplicons were purified with Vazyme VAHTSTM DNA Clean Beads (Vazyme, Nanjing, China) and quantified using the Quant-iT PicoGreen dsDNA Assay Kit (Invitrogen, Carlsbad, CA, USA). After the individual quantification step, amplicons were pooled in equal amounts, and pair-end 2*250 bp sequencing was performed using the Illumina NovaSeq platform with NovaSeq 6000 SP Reagent Kit at Genekinder Medicaltech (Shanghai) Co., Ltd, China.

### 2.3 Analysis of 16S rRNA sequencing data

The 16S rRNA sequencing data were analyzed using the R package DADA2 (v1.16.0), phyloseq (v1.42.0), and microbiome (v1.20.0) according to the official recommended tutorials. Briefly, raw sequence data were first processed using the software cutadapt (v1.18) to remove primer sequences (Martin, 2011), and were then quality filtered, denoised, merged, chimer removed, and annotated using the DADA2 package (Callahan et al., 2016). The microbial reference database was the SILVA Release 132. The processed data and sample information were stored in a phyloseq object. The microbial taxa with fewer than 3 reads were regarded as false positives and then discarded. The microbiome analysis, including diversity analysis, composition analysis, and association analysis, was performed using the package microbiome. LEfSe (linear discriminant analysis effect size) was performed to detect differentially abundant taxa across groups using the default parameters. Microbial functions were predicted by PICRUSt2 (phylogenetic investigation of communities by reconstruction of unobserved states) upon MetaCyc (https://metacyc.org/) (Douglas et al., 2020). Modeling analysis was applied by discriminating the samples from different groups using the R package tidyverse (v2.0.0), which includes a set of modeling and machine learning packages. In this study, seven modeling methods were used: decision tree, logistic regression, multi-layer perceptron (MLP), naïve Bayes, nearest neighbor, random forest, and support vector machine (SVM). The significance of differentiation of microbial communities and other quantitative indexes among groups was assessed by adonis2 (permutational multivariate analysis of variance using distance matrices) and Wilcox rank-sum test. Finally, the visualization of all the results was performed using the package ggplot2 and other relevant packages.

### 2.4 RNA extraction and sequencing

Total RNA was isolated using the Trizol Reagent (Invitrogen Life Technologies), after which the concentration, quality, and integrity were determined using a NanoDrop spectrophotometer (Thermo Scientific). Quality and integrity information is shown in **Supplementary Table S1**. Three micrograms of RNA were used as input material for the RNA sample preparations. Sequencing libraries were generated according to the following steps. Firstly, mRNA was purified from total RNA using poly-T oligo-attached magnetic beads. Fragmentation was carried out using divalent cations under elevated temperature in an Illumina proprietary fragmentation buffer. First strand cDNA was synthesized using random oligonucleotides and Super Script II. Second strand cDNA synthesis was subsequently performed using DNA Polymerase I and RNase H. Remaining overhangs were converted into blunt ends via exonuclease/polymerase activities, and the enzymes were removed. After adenylation of the 3′ ends of the DNA fragments, Illumina PE adapter oligonucleotides were ligated to prepare for hybridization. To select cDNA fragments of the preferred 400–500 bp in length, the library fragments were purified using the AMPure XP system (Beckman Coulter, Beverly, CA, USA). DNA fragments with ligated adaptor molecules on both ends were selectively enriched using Illumina PCR Primer Cocktail in a 15-cycle PCR reaction. Products were purified (AMPure XP system) and quantified using the Agilent high-sensitivity DNA assay on a Bioanalyzer 2100 system (Agilent). The sequencing library was then sequenced on NovaSeq 6000 platform (Illumina) [Genekinder Medicaltech (Shanghai) Co., Ltd, China].

### 2.5 Analysis of RNA-seq data

The raw RNA-Seq data was subjected to the nf-core/rnaseq pipeline for quality control, alignment, and gene expression quantification (Ewels et al., 2020). The version of human reference genome is GRCh38. The raw count matrix was then analyzed by the R package DESeq2 to obtain the differentially expressed genes with the thresholds: |log2FoldChange|>2 and p.adjust <0.05. The package clusterProfiler was used to perform functional enrichment analysis, including gene ontology term enrichment analysis, reactome pathway term enrichment analysis, and gene set enrichment analysis (Wu et al., 2021b). In addition, CIBERSORT was used for the immune cell analysis of the gene expression data with TPM values (Newman et al., 2015). The running parameters were set as follows: relative and absolute modes together, LM22 signature gene file, 100 permutations, and quantile normalization disabled.

### 2.6 Correlation analysis between microbiome and transcriptome data

The correlation analysis was performed between tissue microbiome abundance data at the different taxonomical level and the gene expression data, with the Spearman rank correlation metric performed using the package microbiome (version 1.20.0), vegan (version 2.6-4), and psych (version 2.3.6). Only the significant correlation pairs were kept. Among them, the pairs with significant differential genus and genes were highlighted.

### 2.7 Data decontamination

To ensure the reliability of our microbial analyses, we implemented stringent contamination controls: (1) PCR negative controls (ddH_2_O) confirmed the absence of reagent contamination (reads <1000; no amplification bands); (2) sterile sample collection and environmental controls (validated by electrophoresis) minimized exogenous DNA; (3) bioinformatics filtering excluded taxa with ≤3 reads or unverified by the mbodymap database. Importantly, *Planococcus donghaensis*—validated via FISH and functional assays—exhibited pro-tumorigenic effects, supporting its biological relevance beyond technical artifacts (**Supplementary Figure S2**).

### 2.8 Statistics and reproducibility

All analyses were performed using R software v4.2.0 (https://cran.r-project.org/). Wilcoxon rank-sum test was used to compare the distributions of continuous measurements between two groups. Differential gene expression, GSEA enrichment analyses, and KEGG analyses were subjected to multiple testing adjustments using the Benjamini–Hochberg False Discovery Rate method. Unless otherwise noted, a p.adj-value <0.05 was considered statistically significant.

## 3 Results

### 3.1 PTC tissue indeed harbors intratumoral microbes

**Figure 1:**
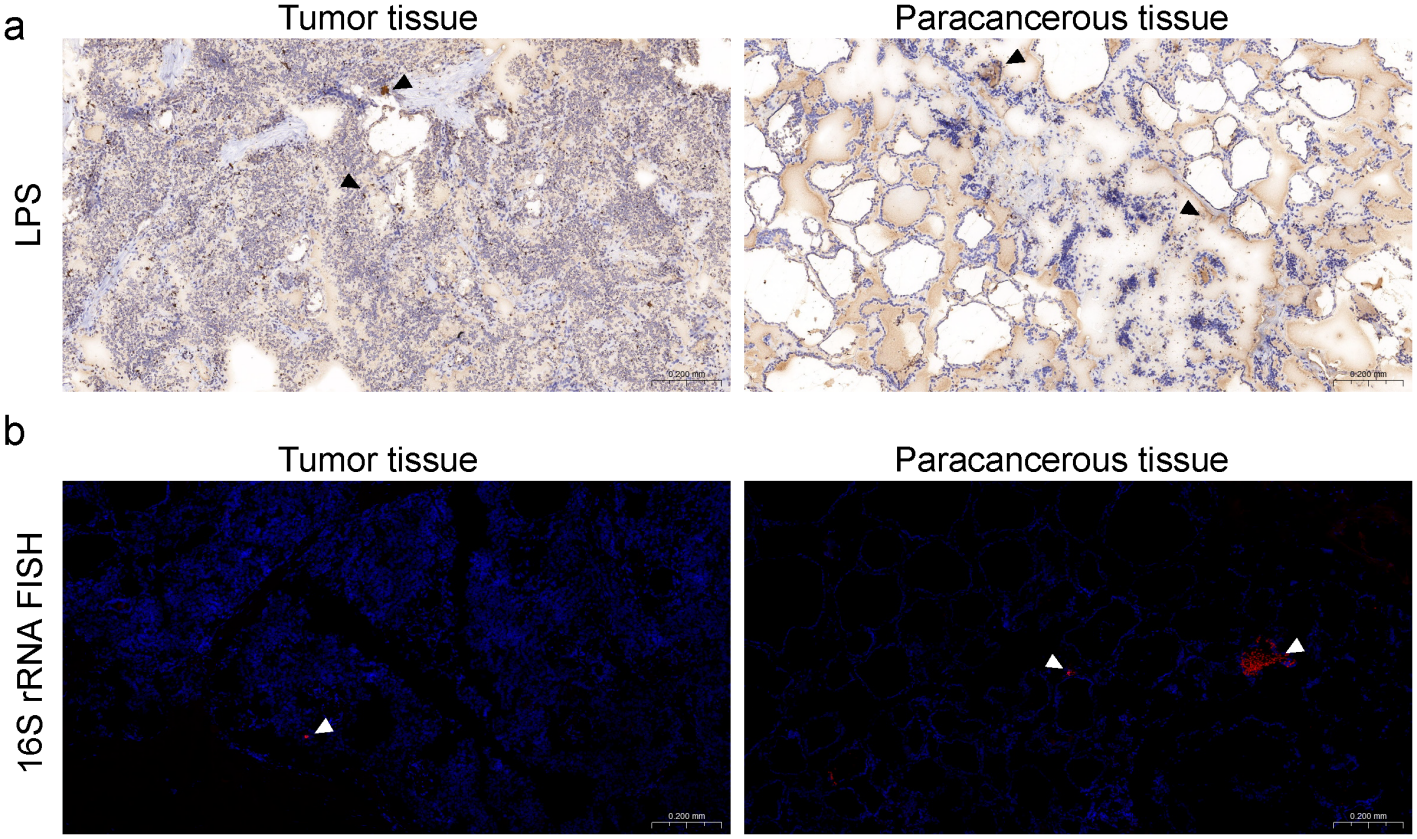
Bacterial components are detected in human PTC tumor and para-cancerous tissues. **(a)** Immunohistochemistry (IHC) of lipopolysaccharide (LPS), black arrows denoting bacteria in immunohistochemical staining. **(b)** 16S rRNA FISH showing bacterial invasion into PTC tumor and para-cancerous tissues, scale bars for dimensional reference; white arrows identifying bacterial signals in FISH analysis.

Although some studies reported the existence of abundant microbes colonized in the TC tissues based on sequencing methods, there is still a lack of traditional experimental evidence to prove the existence of bacteria in TC tissue samples. Therefore, we stained the tumor and para-cancerous tissue of one PTC patient. We performed immunohistochemistry (IHC) staining against bacterial lipopolysaccharide (LPS), which is specific to detecting Gram-negative bacteria (**Figure 1a**). With a universal probe against bacterial 16S rRNA, we also adopted RNA fluorescence *in situ* hybridization (FISH) for detecting bacterial RNA in PTC tissues (**Figure 1b**). Positive 16S rRNA and LPS staining results were observed, indicating the actual presence of bacteria in PTC.

### 3.2 Different microbial communities between tumor and para-cancerous tissue in PTC

In order to reveal the microbial composition of PTC tissue, 16S rRNA amplicon sequencing was performed on the tumor and para-cancerous tissues in 38 PTC patients. The detailed information is listed in **Table 1**.

**Table 1:** Clinical characteristics of the studied PTC patients.

Patient Characteristics	Total Number (n=38)	Percent (%)
Sex		
Male	11	28.9
Female	27	71.1
Age		
Young (<55)	26	68.4
Old (≥55)	12	31.6
Lesions		
Single	25	65.8
Multiple	13	34.2
Gland lobes		
Unilateral	29	76.3
Both	9	23.7
Capsular invasion		
Yes	14	36.8
No	24	63.2
T-stage		
T1a	28	73.7
T1b	10	26.3
N-stage		
N0	13	34.2
N1a	23	60.5
N1b	2	5.3
M-stage		
M0	38	100
pTNM		
I	31	81.6
II	7	18.4
ATA risk stratification		
Low	36	94.7
Intermediate	2	5.3

**Figure 2:**
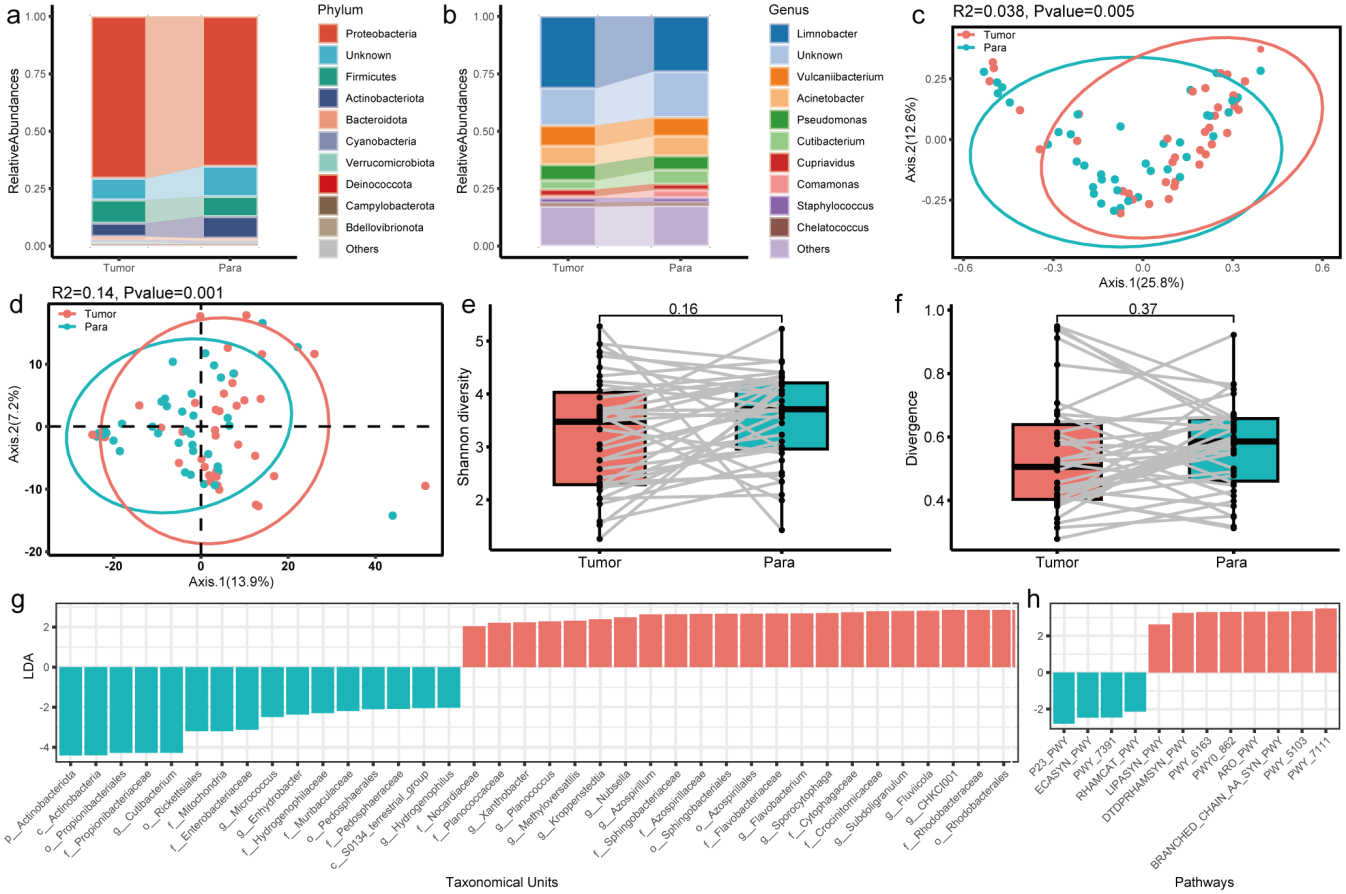
Profile of the intratumoral microbiome between tumor and para-cancerous tissue in PTC patients. **(a, b)** Top 10 phyla and genera of the microbiome for the tumor and para-cancerous tissue. **(c, d)** PCoA analysis of microbial composition and predicted functional composition based on the Bray–Curtis distance colored by sample type. **(e, f)** The comparison of alpha diversity estimated using Shannon index and divergence between tumor and para-cancerous tissue in PTC. **(g, h)** LEfSe analysis identified the microbes and pathways whose abundances significantly differed between tumor and para-cancerous tissue in PTC.

After quality control, a total of 861 genera and 43 phyla were identified according to the phylogenetic taxonomical levels. The three predominant phyla in each group were *Proteobacteria*, *Firmicutes*, and *Actinobacteria*, altogether contributing up to 84.9% of the tissue microbial community on average (**Figure 2a**). The average compositions of the microbial communities at the genus level are shown in **Figure 2b**, of which *Limnobacter*, *Vulcaniibacterium*, and *Acinetobacter* occupied the top 3. PCoA plot evaluated by Bray–Curtis distances revealed a significant distinction of tissue microbiota between para and tumor samples in PTC (**Figure 2c**, adonis2 test, p-value=0.005, R2 = 0.038). A similar result was observed in the predicted microbial pathway distribution of tissue microbiota by PICRUSt2 (**Figure 2d**, adonis2 test, p-value=0.001, R2 = 0.14).

In order to compare the differences in microbial communities between tumor and para-cancerous samples, diversity and differential analysis were further conducted. The alpha diversity (Shannon index, **Figure 2e**) and divergence level (**Figure 2f**) in para-cancerous tissues were slightly higher than those in tumor tissues without significance, suggesting a convergent tendency of tissue microbiome after oncogenesis. Other diversity indices including diversity_gini_simpson, evenness_camargo, and rarity_rare_abundance presented similar results (**Supplementary Figure S3**). Moreover, we identified 22 and 16 taxonomic units significantly overrepresented in tumor and para-cancerous tissues, respectively (**Figure 2g**). *Clostridiales bacterium CHKCI001* was the most enriched genus in tumor, which was one kind of proinflammatory bacteria commonly located in the gut. It was reported that *Clostridiales* had the cancer-promoting and anticancer activities via different ways (Montalban-Arques et al., 2021; Wu et al., 2021a). On the contrary, *Cutibacterium* was the most enriched genus in para-cancerous tissue, which was reported as a common member of skin microbiota. It was found to be prevalent in thyroid cancer samples and to be associated with immune suppression and poor prognosis in a subpopulation of thyroid cancer (Trivedi et al., 2023).

Moreover, the differential enriched pathways between tumor and para-cancerous tissues were also identified (**Figure 2h**). Among them, the pathways involved in pyruvate fermentation to isobutanol (PWY-7111), L-isoleucine biosynthesis III (PWY-5103), and superpathway of branched amino acid (BRANCHED_CHAIN_AA_SYN_PWY), were the top 3 enriched pathways in tumor tissues (**Figure 2h**), which were also reported to be enriched in the GC samples compared to superficial gastritis and atrophic gastritis (Huang et al., 2021). In contrast, an enrichment of the synthesis pathways, including the reductive TCA cycle pathway (P23-PWY), enterobacterial common antigen biosynthesis (ECASYN-PWY), and isoprene biosynthesis II (PWY- 7391) (**Figure 2h**), were found in para-cancerous tissues. This demonstrates that the taxonomic differences observed between tumor and normal tissues resulted in different microbiome functionality.

### 3.3 The microbial communities vary as the PTC progresses

**Figure 3:**
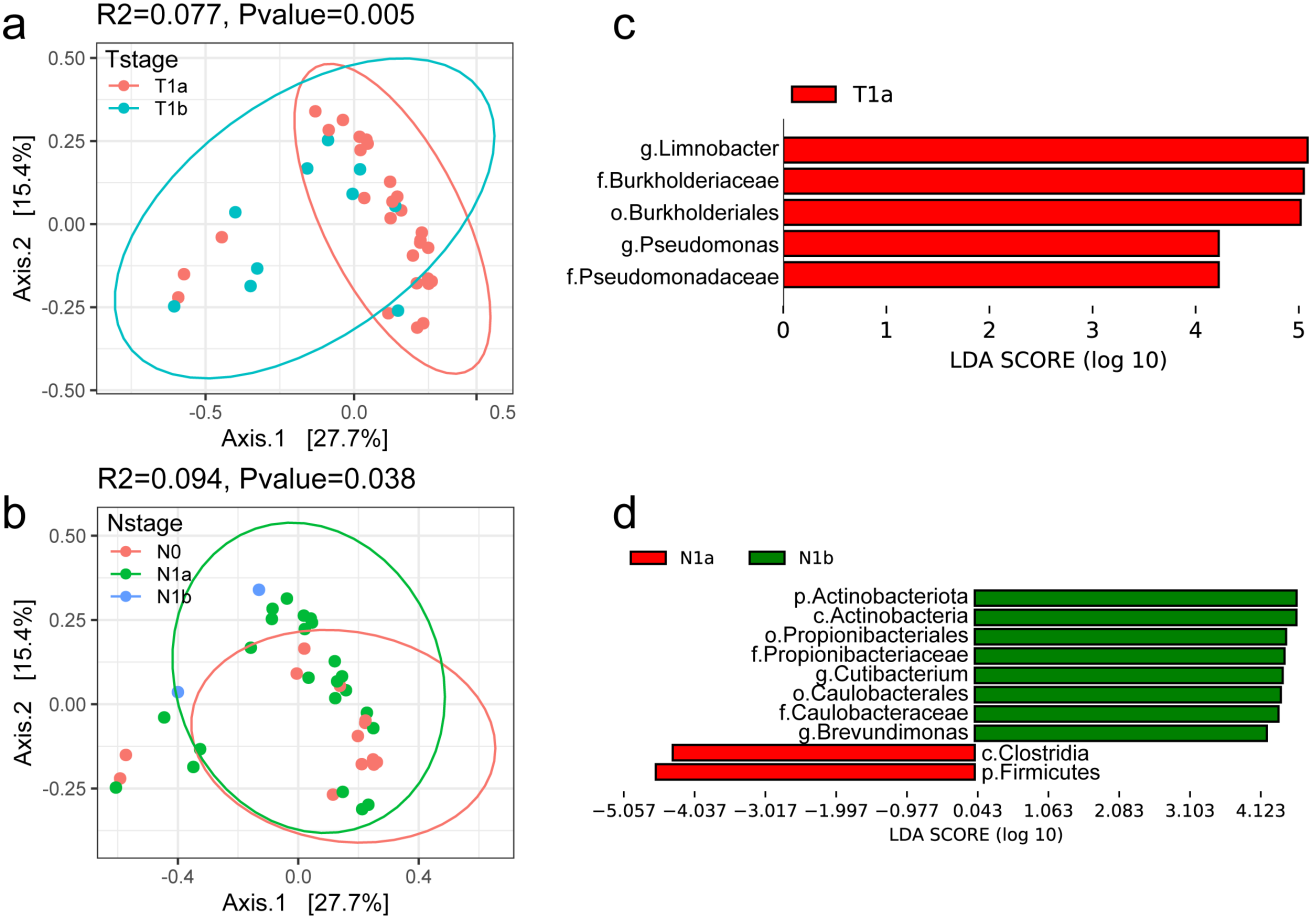
Profile of the intratumoral microbiome in different clinical stages. **(a, b)** PCoA analysis of microbial composition based on the Bray–Curtis divergence colored by T-stage and N-stage. **(c, d)** Bar plot of differential microbial taxa at the genus level between T-stage and N-stage.

It was reported that the tumor microbiome communities are significantly associated with tumor invasion in patients with resected PTC. Therefore, the general landscape of the tumor microbiome composition was assessed with the clinical indexes in this study. We found that T-stage and N-stage had significant effects on the microbial compositions in the tumor microenvironment (**Figures 3a, b**). Notably, the R2 values (proportion of variance explained by categorical grouping) were higher in the two factors compared to the factor sample type (tumor vs. normal). The factor N-stage had the highest R2 values, while the T-stage had the most obvious significance. These results suggest a continuous change in the microbial communities as the PTC progresses.

The differential taxa in the two sets of comparison were further identified. The differential genera with LDA > 4 are shown in **Figure 3c, d**. Among them, *Limnobacter* was significantly enriched in T1a and reduced in T1b stage. It was reported as one of the potential biomarkers for hepatocellular carcinoma (Zheng et al., 2020) and lung cancer (Zheng et al., 2023). *Pseudomonas* was the other genus with higher abundance in T1a than that in T1b, which is consistent with previous results in Yuan L’s study (Yuan et al., 2022).

We also found that the abundance of *Cutibacterium* and *Brevundimonas* showed an increase as the N-stage increases. The genus *Cutibacterium* was reported to be prevalent in thyroid cancer samples and was found to be associated with immune suppression and poor survival (Trivedi et al., 2023). *Brevundimonas* is a genus of Gram-negative bacteria widely distributed in nature and is also an opportunistic pathogen causing healthcare-associated infections (Liu et al., 2021). However, its role in tumorigenesis needed further and deep investigation. Furthermore, we developed predictive models using multiple machine learning approaches based on the microbial profiles. The results suggest that tissue-associated microbiota may serve as potential biomarkers for tumor classification and staging, demonstrating promising clinical applicability (**Supplementary Figure S4**).

### 3.4 Global overview of PTC transcriptome

**Figure 4:**
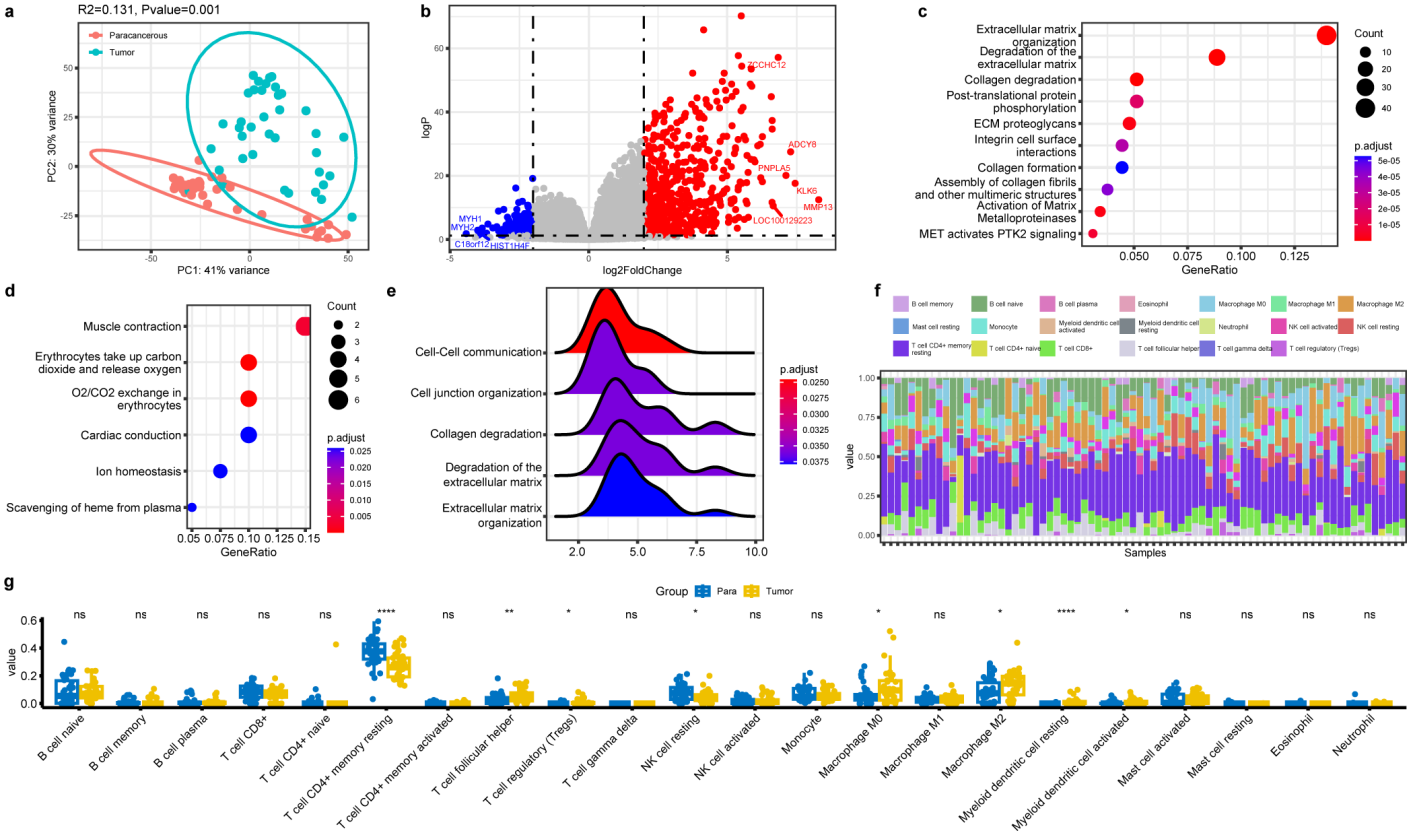
Profile of host gene expression between the tumor tissue and para-cancerous tissues in PTC. **(a)** PCA analysis of host transcriptome based on similarities between samples. **(b)** Volcano plot of differential expressed genes in the tumor tissues. **(c, d)** Dot plot of enriched reactome pathways of downregulated and upregulated DEGs. **(e)** GSEA analysis indicating ECM-related pathways that are differentially regulated. **(f)** Immune cell composition of all PTC samples identified by the algorithm CIBERSORT. **(g)** Composition comparison of the immune cell types in the tumor and para-cancerous tissues. Significance was labeled with stars (*): * p<0.05, ** p<0.01, **** p<0.0001, ns, not significant.

Since PTC patients demonstrated the presence of microbiota and their associations with tumorigenesis and progression, we hypothesized that changes in the PTC tumorigenesis transcriptome may be correlated with the thyroid tissue microbiome. Therefore, the paired tumor and para-cancerous tissues were subjected to RNA-Seq. The principal component analysis (PCA) plots showed that the expression profile of PTC tumor tissue samples was distinct from that of the para-cancerous tissue samples (**Figure 4a**). After the standard transcriptome analysis, we identified 793 differentially expressed genes (DEGs), which included 617 upregulated and 176 downregulated genes in tumor tissue (**Figure 4b**). *MMP13* was the most upregulated gene, which is a member of the endopeptidase matrix metalloproteinase family and involved in many normal physiological processes. A recent study showed that *MMP13* is often overexpressed across cancer and predicts poor prognosis (Zhang et al., 2023). On the contrary, the expression of *MYH2* had the most obvious downregulation, which was proved as a marker in distinguishing head and neck squamous cell carcinoma and lung squamous cell carcinoma (Vachani et al., 2007). Pathway enrichment analysis showed that upregulated DEGs were mainly enriched in cell extracellular related pathways (**Figure 4c**), such as extracellular matrix organization and integrin cell surface interactions, which play pivotal roles in cancer proliferation, survival, and invasion. The downregulated DEGs were enriched in muscle contraction and o2/co2 exchange in erythrocytes (**Figure 4d**). Moreover, GSEA analysis of these DEGs showed enrichment of cell–cell communication, cell junction organization, collagen degradation, degradation of the extracellular matrix, and extracellular matrix organization (**Figure 4e**).

Besides, the immunological cell types in the tissue were determined from the transcriptome data using the CIBERSORT algorithm (**Figure 4f**). Among them, T cell CD4+ memory resting took the dominant role in thyroid tissue and showed a significant decrease in tumor compared to the para-cancerous tissue (**Figure 4g**). NK cell resting also present the similar trend. The other cell types with significant differences included T cell follicular helper, Tregs, macrophage M0, macrophage M2, myeloid dendritic cell resting, and myeloid dendritic cell activated, all of which showed significant increases in tumor compared to those in normal tissue. Among them, the M0 and M2 macrophages could increase cancer invasion ability, and the Tregs could increase the ability of the cancer cells to escape the immune system and foster cancer progression (Lainé et al., 2021). In a word, increase in the six types of cells within the tumor tissue implied their involvement in pathogenesis and tumor progression.

Notably, there were also significant differences in different clinical groups based on T-stage and N-stage (**Supplementary Figure S5**), suggesting continuous changes as tumor progressed from Stage I to II, although they were all early-stage PTC.

### 3.5 PTC transcriptome profile influenced by the tissue microbiota

**Figure 5:**
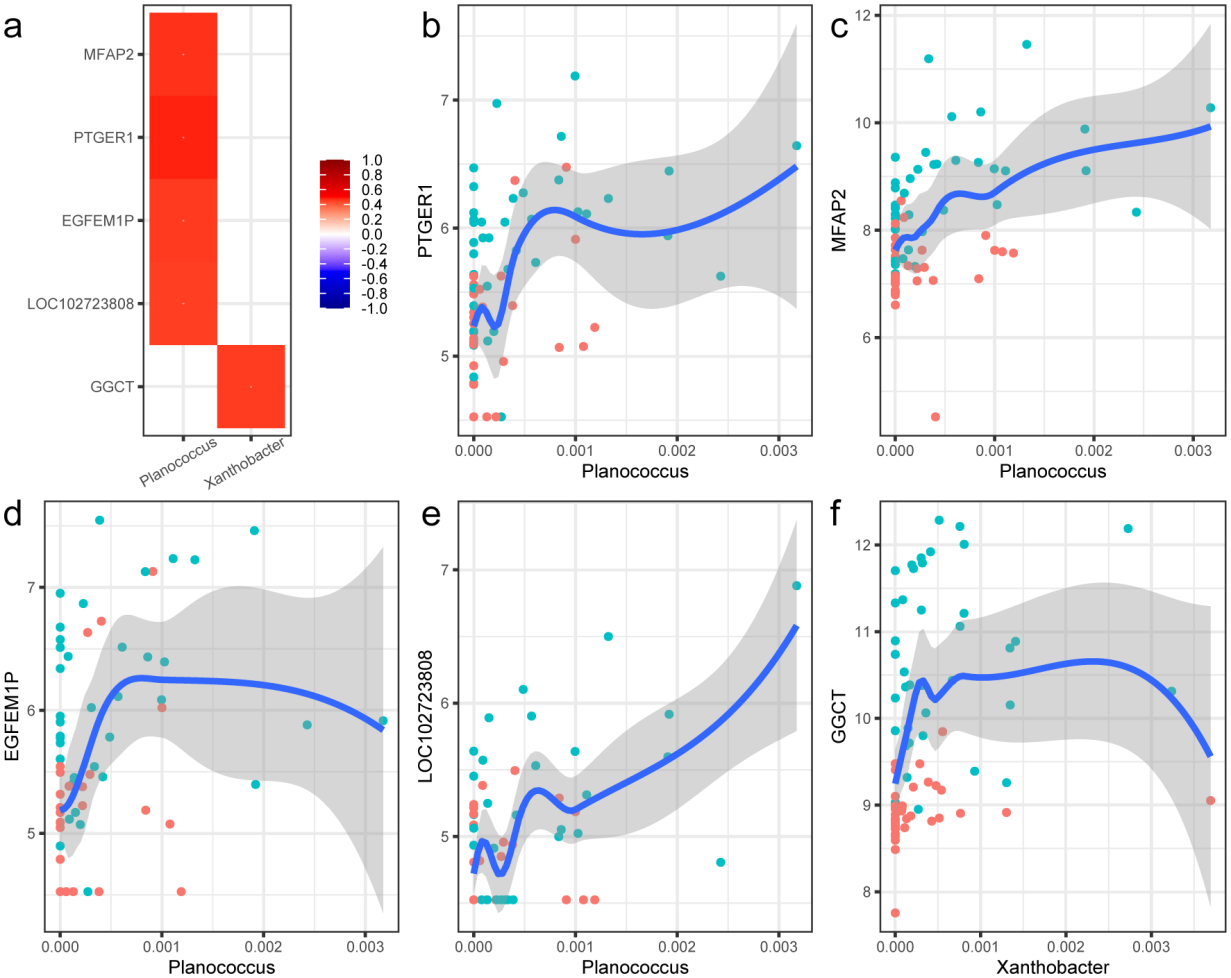
Correlations between the special bacterial taxa and host genes. **(a)** Heat map of the correlations between the two differential microbial genus and five DEGs. The correlations were evaluated with the Spearman method. The correlation coefficients are shown in the legend, and p-values were all less than 0.05. **(b–f)** Nonlinear fitting curve of the abundance of genus *Phlanococcus* and the expression of gene *PTGER1*, *MFAP2*, *EGFEM1P*, *LOC102723808*, and *GGCT*. Each point in the figure stands for a sample. Blue indicates tumor tissue samples, and red represents the para-cancerous tissue samples.

Based on the above-described microbiota and transcriptome data, Spearman’s correlation-based analysis was performed to discover microbe-associated genes and to test whether host transcriptional profiles in PTC could be partially influenced by tissue microbiota. A total of 802 genera and 4896 genes were identified, forming 29,659 genus–gene pairs. However, among them, only two differential genera in abundance and five differential expression genes mentioned above were filtered out (**Figure 5a**). All the pairs showed positive correlations, and the genus and genes were all enriched in the tumor tissues. The strongest correlation pair was the genus *Planococcus* and *PTGER1*, the former of which also showed positive correlation with the other three genes, including *MFAP2*, *EGFEM1P*, and *LOC102723808*. The detailed fitting relationships are presented in **Figures 5b–e**. The genus *Planococcus* is a halophilic bacterium known for the production of diverse secondary metabolites, which was reported to be associated with the stomach neoplasms according to the database GMrepo (Dai et al., 2022). Notably, among the genes, *MFAP2*, encoding the Microfibril Associated Protein 2, was reported to be involved in tumor cell invasion and metastasis (Xu et al., 2022). The downregulation of *MFAP2* could inhibit BCPAP and TPC-1 cell migration and invasion and lymph node metastasis in thyroid papillary carcinoma (Dong et al., 2020). *EGFEM1P* was also reported to be upregulated in papillary thyroid tumors and thyroid cancer cells compared with normal adjacent tissues, and promoted thyroid cancer progression by acting as an miR-369-3p sponge and upregulation TCF4 (Yi et al., 2022). Another pair was the genus *Xanthobacter* and gene *GGCT* (**Figure 5f**). *Xanthobacter* was regarded an associative N2-fixing bacterium and rarely reported in human body. However, the correlated gene *GGCT* was reported to be highly expressed in PTC tissue and cell lines and could promote cell proliferation and migration by activating the MAPK/ERK pathway (Zhang et al., 2022).

### 3.6 PTC immunological profile influenced by the tissue microbiota

**Figure 6:**
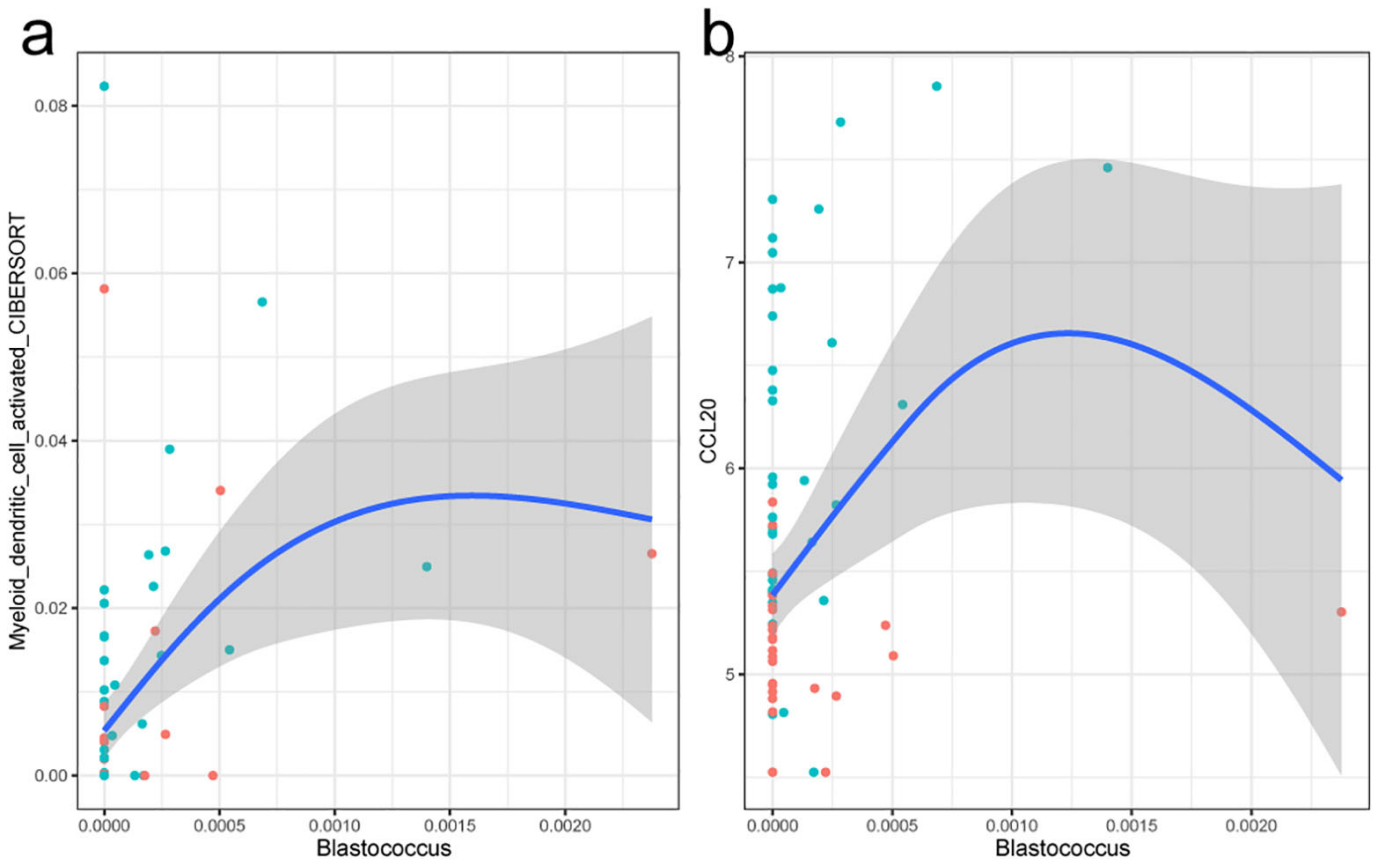
Fitting curve of the abundance of the myeloid dendritic cell activated with genus *Blastcoccus* **(a)** and expression of gene CCL20 **(b)**. Each point in the figure stands for a sample. Blue indicates tumor tissue samples, and red represents the para-cancerous tissue samples.

Considering the differences of immune cell compositions between tumor and para-cancerous tissue in PTC, we further sought to detect whether the tumor tissue microbiome influenced the immune cells in tumor environment. The Spearman’s correlation analysis was also performed between the microbial and expression profiles. A total of eight genus and three cell types were identified, forming eight genus–cell pairs. Among them, no significant differential genus but one differential cell type (myeloid dendritic cell activated) was included. The genus *Blastococcus* was positively related with the myeloid dendritic cell activated (**Figure 6**). *Blastococcus* is Gram-positive, coccoid units, often reproducing by budding and multiple fission, giving rise to a variety of cell forms and aggregates, which mainly colonized various nature environments. However, a recent study showed that the genus *Blastococcus* could be transferred to the skin and respiratory tract of humans after green space exposure (Selway et al., 2020). Myeloid dendritic cells (mDCs) comprise a heterogeneous population of professional antigen-presenting cells, which are responsible for the capture, processing, and presentation of antigens on their surface to T cells. mDCs recruitment into the TME has been reported to depend on the CCR6/CCL20 axis, the latter of which showed a significant increase of expression in tumor tissues (logFC=3.5). In this way, a possible hypothesis might be that the induction of *Blastococcus* would drive the tumor progression by the increased expression of *CCL20* and mDCs.

## 4 Discussion

In recent years, a series of studies have found that there are a large number of microorganisms in tumor tissue and that some of these microorganisms are involved in tumor initiation and development (Xue et al., 2023). In this study, we explored the role of intratumoral microbiome composition in PTC and its association with the host transcriptome. Overall, we observed a substantial microbiota presence in the PTCs of all patients. And the microbial communities between the tumor and para-cancerous tissues showed significant differences. The tissue microbiome changed as the tumor progressed. We also observed the significant differences in transcriptome and immune cell composition between the tumor tissue and para-cancerous tissue. A set of differential microbes, differentially expressed genes, and differential immune cells were then identified. And the influences of microbiota on the host gene expression and immune cells were finally determined using the Spearman’s rank correlation test. In a word, these results not only validated the presence of bacteria in thyroid tissue but also suggested the potential interactions of microbe–gene and microbe–cell in tumorigenesis and tumor progression.

Growing studies have revealed the possible effects of intratumoral microorganisms on the occurrence and development of tumors. In this study, we also identified a set of significantly differential microbes associated with tumor status. Among them, 11 genera, including *Clostridiales bacterium CHKCI001*, *Fluviicola*, *Subdoligranulum*, *Sporocytophaga*, *Flavobacterium*, *Azospirillum*, *Nubsella*, *Kroppenstedtia*, *Methyloversatilis*, *Planococcus*, and *Xanthobacter*, were significantly enriched in tumor tissue, while 4 genera *Hydrogenophilus*, *Enhydrobacter*, *Micrococcus*, and *Cutibacterium* were enriched in the para-cancerous tissues. According to the curated database mbodymap (Jin et al., 2021), all these genera were almost detected in human body and were mainly prevalent in the lung and upper respiratory tract whether the individual was sick or healthy. According to the database, *Fluviicola*, *Microcossus*, and *Cutibacterium* were labeled as a marker for ovarian neoplasms. *Flavobacterium* was also labeled as a positive marker for endometrial neoplasms. *Azospirillum*, *Flavobacterium*, and *Cutibacterium* were associated with Alzheimer disease and cognitive dysfunctions. *Micrococcus* and *Planococcus* were negatively related with atopic dermatitis. In a word, these microbes are part of the human microbiome, and may play an important role in the development of various diseases.

Though we discovered some specific microbial signatures in PTC, little was known about the role and mechanisms of these microbes on the pathogenesis and progression. We identified a set of signatures of cell-associated bacteria and host-gene-associated bacteria using the correlation analysis to derive potential causality indirectly, which were then summarized as a microbe–gene–cell interaction network (**Supplementary Figure S6**). We characterized the tumor-enriched bacteria *Planococcus* and *Xanthobacter* as the potential PTC-promoting bacteria that affect the host transcriptome of PTC. *Planococcus*, a halophilic bacterium, was known to produce the biosurfactants, which could act as antibacterial, anticancer activity (Waghmode et al., 2020). It was found to be enriched in circulation and play a role in inflammation in systemic lupus erythematosus (Cheng et al., 2023). As we known, PTC is classified as an inflammation-driven cancer, and the immune system is enhanced during the occurrence and development of PTC. Its associated genes, which were highlighted in the above results, were involved in various cancers, including PTC. Through further literature research, we found that they all take roles in the host inflammation response. *PTGER1* is one of the four prostaglandin receptors involved in biological processes such as immunity, inflammation, and pain conduction (Tober et al., 2007). *MFAP2* had the anti-inflammation function, and increased levels of *MFAP2* expression were found to be a mechanism triggered to bind excess TGF-beta to control inflammation. As for the lncRNA *EGFEM1P*, it was reported that it promotes thyroid cancer progression by acting as an miR−369−3p sponge and upregulating *TCF4* (Yi et al., 2022). However, the upregulation of miR-369-3p could suppress the LPS-induced inflammatory response, reducing C/EBP-β, TNFα, and IL-6 production (Scalavino et al., 2020). *LOC102723808* was an uncharacterized gene and not well known. In a recent study, it was reported to be significantly upregulated in human lung organoids after SARS-CoV-2 infection (Han et al., 2020). In a word, it could be hypothesized that the colonization or increase of special microbes, such as *Planococcus*, would induce a sustained inflammatory response in the host that promotes the development of PTC.

In addition, the genus *Xanthobacter* and gene *GGCT* had a significantly positive correlation. As we know, *GGCT* encodes the Gamma-glutamylcyclotransferase, one of the major enzymes involved in glutathione metabolism (Kageyama et al., 2015), and was involved in the carcinogenesis and progression of PTC (Li et al., 2022). Interestingly, *Xanthobacter*, an aerobic or facultative anaerobic fermentation Gram-negative bacteria, could contribute to the glutathione metabolism for harboring the relevant functional genes (e.g., nbzAa, catA, CYC, pilA) (Chen et al., 2023). This significant correlation pair suggested the important role of glutathione homeostasis in the pathogenesis and progression of PTC and potential therapeutics for PTC.

Our study has limitations. First, according to the TNM stage, all of the PTC patients were in T1/N0-1/M0 stage, which was in the early stage of PTC. Although there was no data on advanced patients, this study provided an opportunity to reveal profiles and changes in the microbiome and transcriptome in the early-stage PTC. The results revealed that the microbiota exist in the tumor microenvironment and are involved in the tumor occurrence and progression in the early stage of PTC. Second, our study focused on the correlations, not causality. As we know, studying causality is challenging in humans, especially the internal tissue of human body. However, the identified microbial signatures and microbe–host gene interactions provided a set of candidate targets for future *in vivo* and *in vitro* researches. Relevant experimental exploration is currently in progress in our team, and the results also provide references for interested peers. Third, the sample size is not large enough. On the one hand, we preliminarily confirmed the existence of microorganisms through experimental methods (LPS staining). On the other hand, the public databases (mbodymap and GMrepo) and extensive literature research were used to confirm the important and differential microbes.

## Data Availability

The datasets presented in this study can be found in online repositories. The names of the repository/repositories and accession number(s) can be found in the article/**Supplementary Material**.
