# Supplementary figures and images for: Integrated analysis of microbiome and host transcriptome revealed correlations between tissue microbiota and tumor progression in early-stage papillary thyroid carcinoma
Source: Front Cell Infect Microbiol. 2025 Jun 9;15:1571341. doi: 10.3389/fcimb.2025.1571341 (PMC12183207; doi:10.3389/fcimb.2025.1571341)

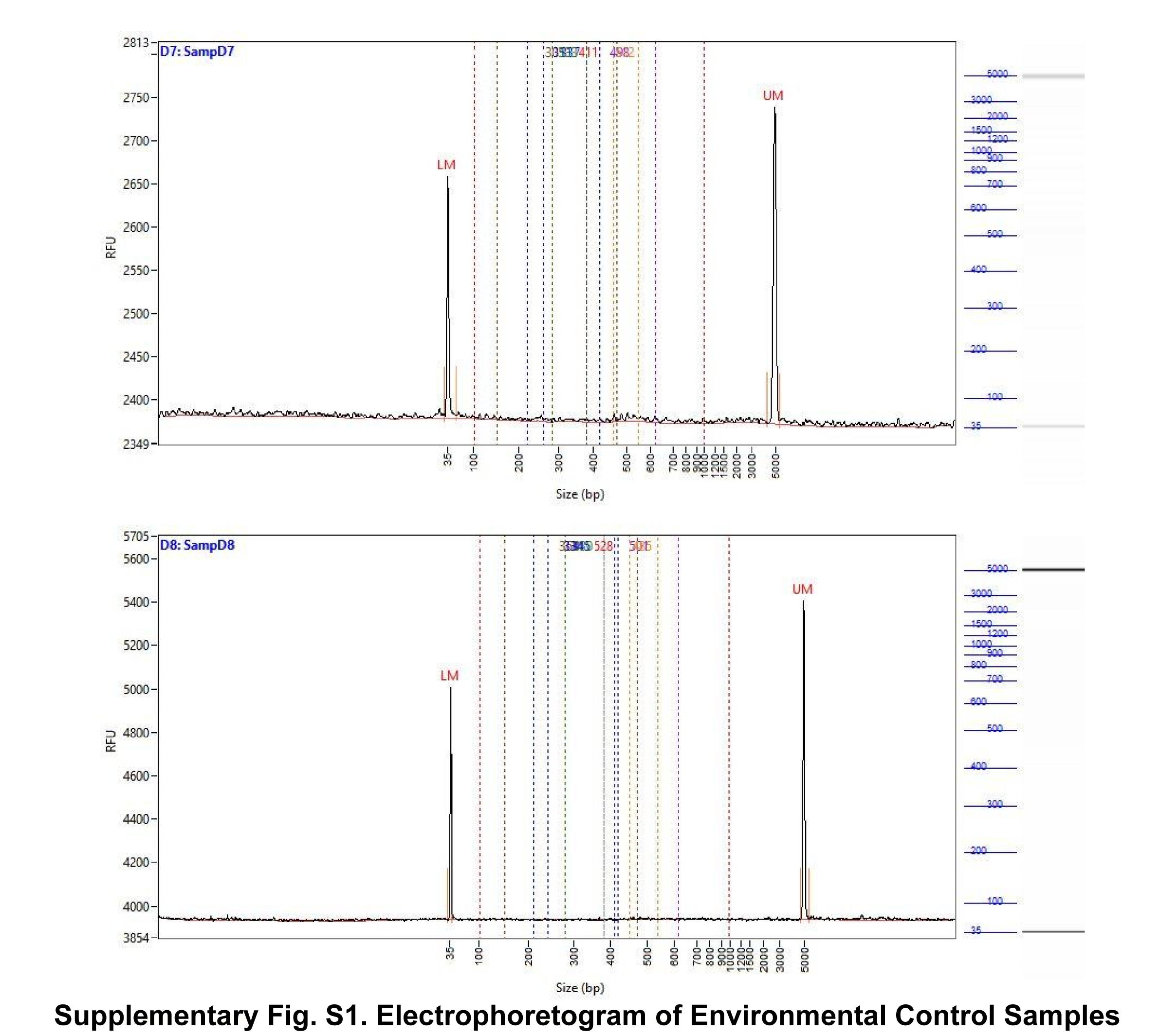

Supplement: Supplementary file 1 [file Image1.tif]

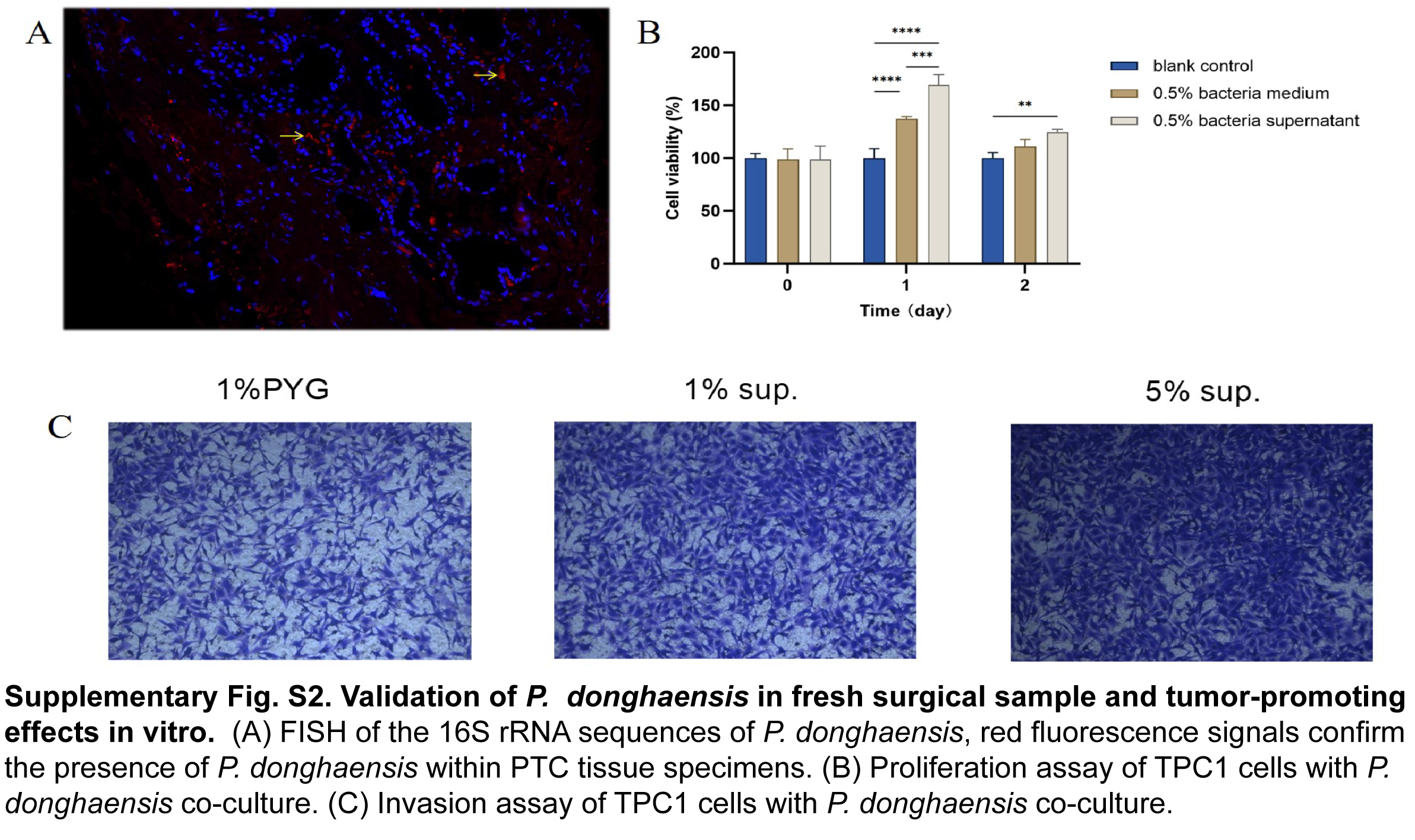

Supplement: Supplementary file 2 [file Image2.tif]

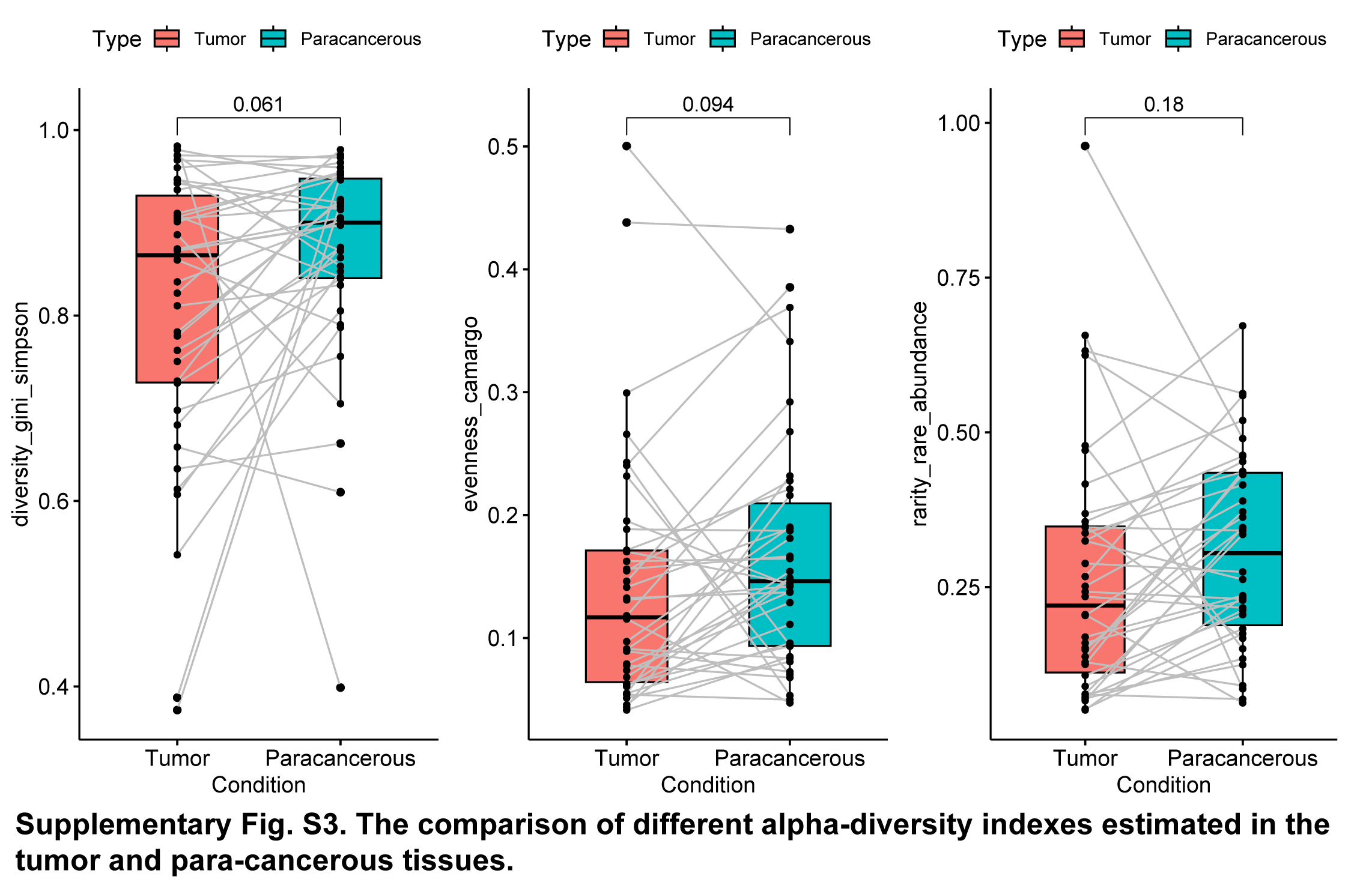

Supplement: Supplementary file 3 [file Image3.tif]

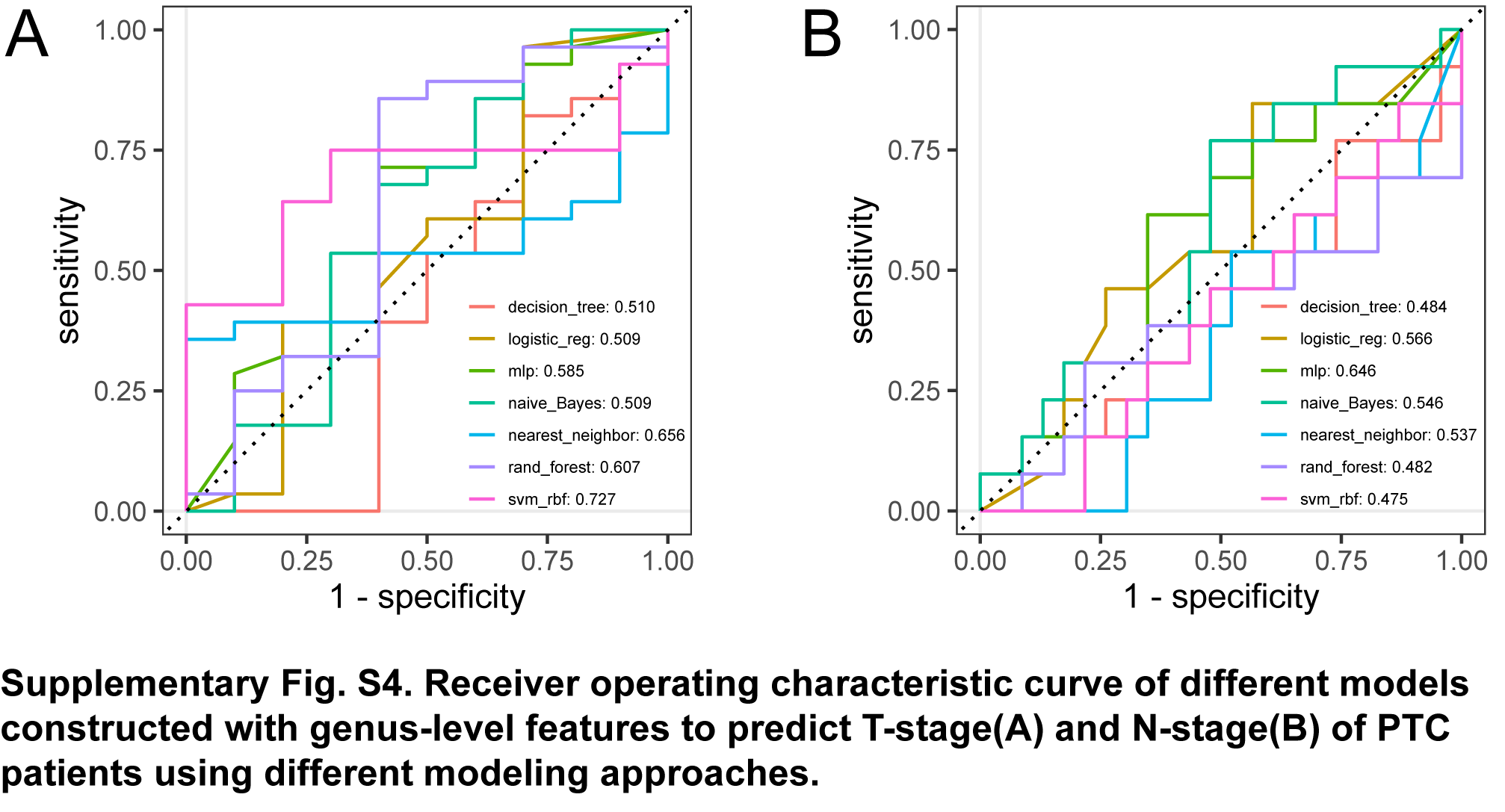

Supplement: Supplementary file 4 [file Image4.tif]

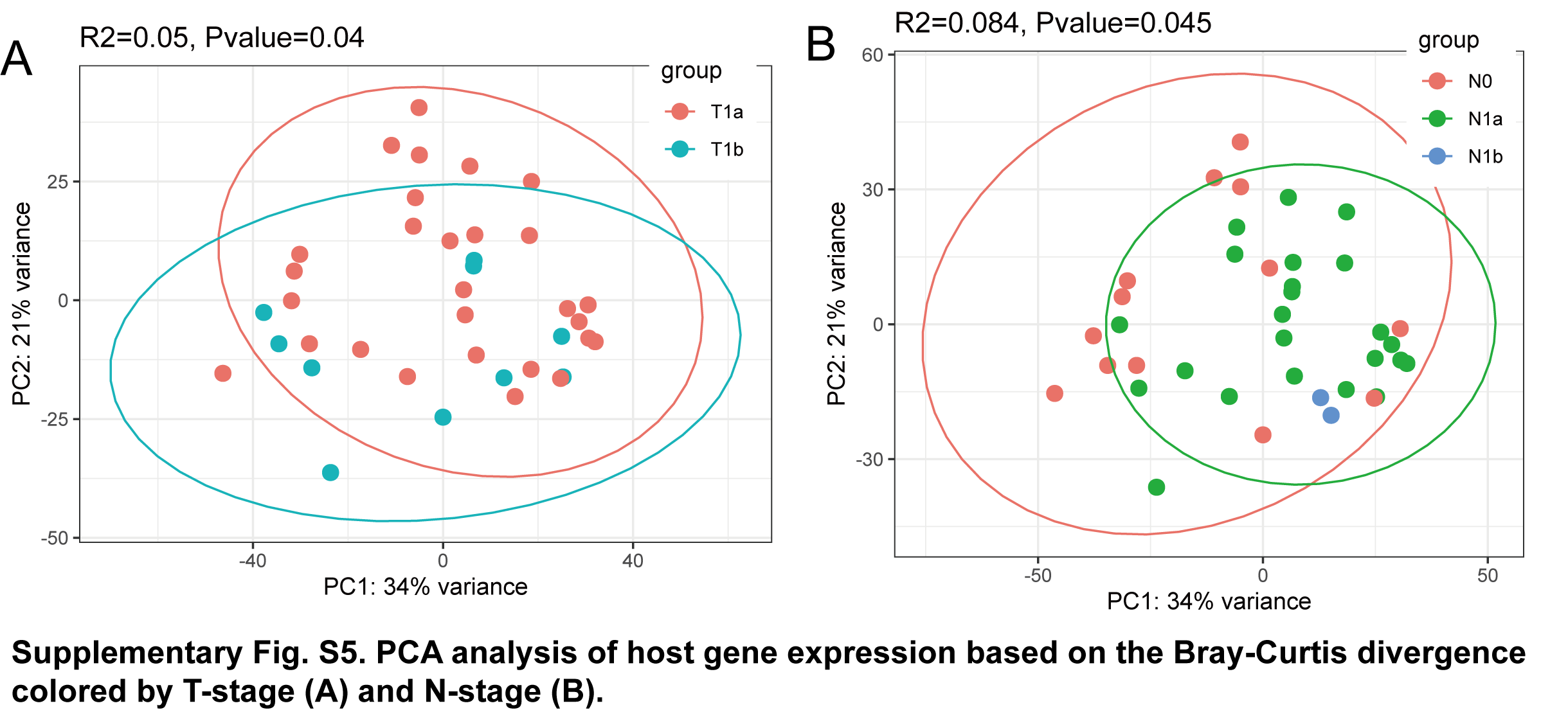

Supplement: Supplementary file 5 [file Image5.tif]

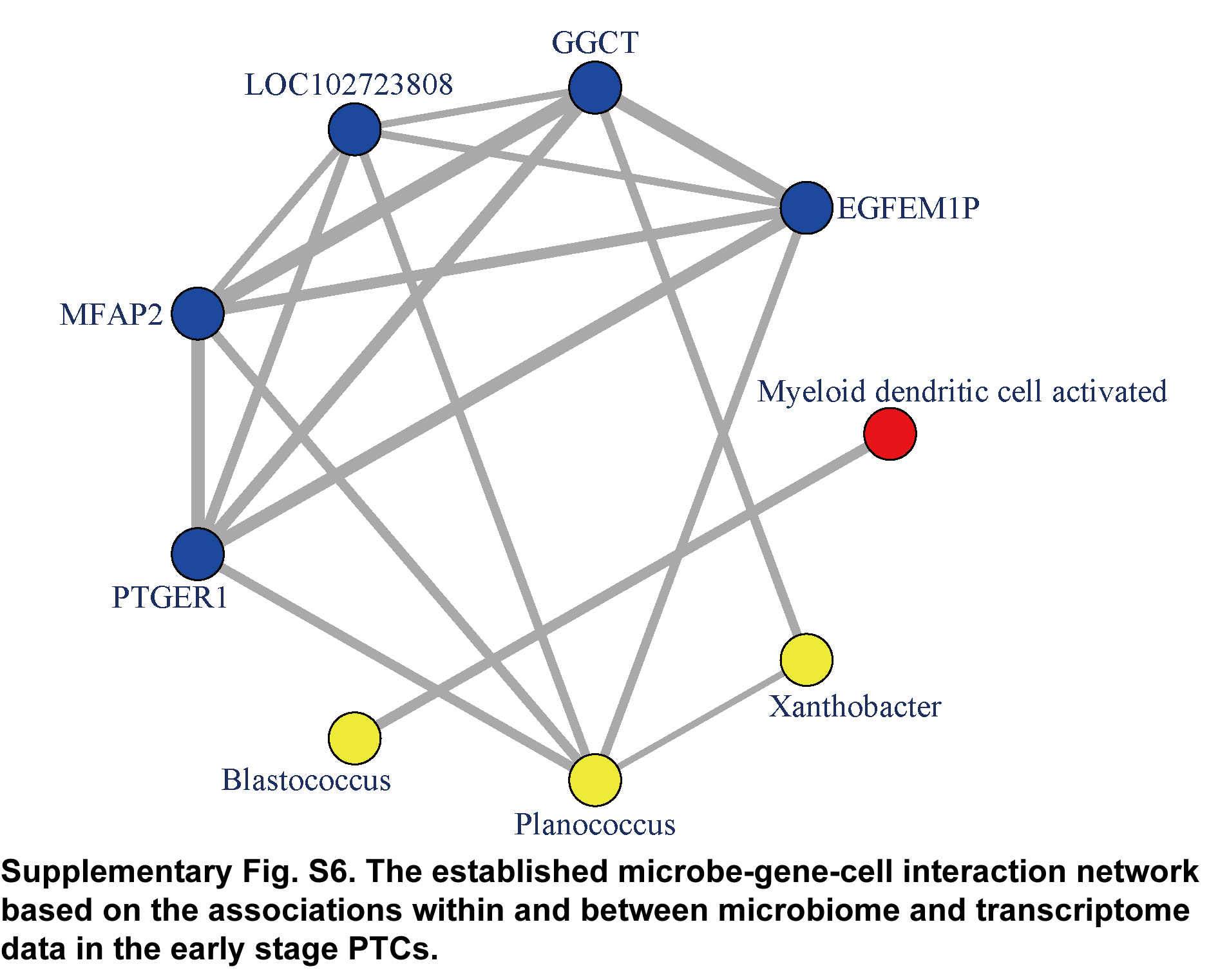

Supplement: Supplementary file 6 [file Image6.tif]
